# Detection of caries lesions using a water-sensitive STIR sequence in dental MRI

**DOI:** 10.1038/s41598-024-51151-2

**Published:** 2024-01-05

**Authors:** Egon Burian, Nicolas Lenhart, Tobias Greve, Jannis Bodden, Gintare Burian, Benjamin Palla, Florian Probst, Monika Probst, Meinrad Beer, Matthias Folwaczny, Julian Schwarting

**Affiliations:** 1 Department of Diagnostic and Interventional Neuroradiology, Klinikum rechts der Isar, School of Medicine, Technical University of Munich, Munich, Germany; 2 Department of Diagnostic and Interventional Radiology, Universitätsklinikum Ulm, Ulm, Germany; 3 Department of Diagnostic and Interventional Neuroradiology, Kantonsspital Frauenfeld, Thurgau AG, Frauenfeld, Switzerland; 4 Department of Diagnostic and Interventional Radiology, Klinikum rechts der Isar, School of Medicine, Technical University of Munich, Munich, Germany; 5 Department of Neurosurgery, University of Munich, Munich, Germany; 6 Department of Prosthodontics, LMU University Hospital, Ludwig-Maximilians-University, Munich, Germany; 7 Department of Oral and Maxillofacial Surgery, University of Illinois, Chicago, Chicago, USA; 8 Department of Oral and Maxillofacial Surgery and Facial Plastic Surgery, University Hospital LMU Munich, 80337 Munich, Germany; 9 Department of Restorative Dentistry and Periodontology, LMU University Hospital, Ludwig-Maximilians-University, Munich, Germany

**Keywords:** Translational research, Acute inflammation, Dental diseases, Dental caries, Pulpitis

## Abstract

In clinical practice, diagnosis of suspected carious lesions is verified by using conventional dental radiography (DR), including panoramic radiography (OPT), bitewing imaging, and dental X-ray. The aim of this study was to evaluate the use of magnetic resonance imaging (MRI) for caries visualization. Fourteen patients with clinically suspected carious lesions, verified by standardized dental examination including DR and OPT, were imaged with 3D isotropic T2-weighted STIR (short tau inversion recovery) and T1 FFE Black bone sequences. Intensities of dental caries, hard tissue and pulp were measured and calculated as aSNR (apparent signal to noise ratio) and aHTMCNR (apparent hard tissue to muscle contrast to noise ratio) in both sequences. Imaging findings were then correlated to clinical examination results. In STIR as well as in T1 FFE black bone images, aSNR and aHTMCNR was significantly higher in carious lesions than in healthy hard tissue (p < 0.001). Using water-sensitive STIR sequence allowed for detecting significantly lower aSNR and aHTMCNR in carious teeth compared to healthy teeth (p = 0.01). The use of MRI for the detection of caries is a promising imaging technique that may complement clinical exams and traditional imaging.

## Introduction

In clinical practice, carious lesions are detected based on inspection, instrumentation, and traditional radiographic imaging such as orthopantomogram (OPT) or bitewing radiographs^1,2^. Recent research has also shown that near-ultraviolet to near-infrared spectrum light can detect caries with increasing utilization in clinical settings^3,4^. Detection of caries by traditional radiographs relies on the demineralization of enamel or dentin, a late stage in the caries process. In the last decade, magnetic resonance imaging (MRI) and investigational sequences have become an attractive alternative in detecting carious lesions^5,6^. MRI could offer two significant benefits: first a reduction in radiation exposure, and second, the detection of carious lesions prior to traditional radiographic signs.

In a 2020 study by Cankar, the authors showed T2 mapping on MRI can detect pulpal reaction from penetration of caries into dentin^7^. In a 2015 systematic review by Schwendicke et al., radiographic caries detection was found more effective in advanced carious lesions, while alternative methods could be considered for initial carious lesions in populations with high caries risk or prevalence^2^. This conclusion highlights the limitation of traditional radiographic techniques, which are only capable of imaging carious lesions after enamel or dentin has been demineralized. The preceding steps, such as saliva penetration into porous tooth surface or reactive tertiary dentine formation, are concealed^8^. In 2001, Ekstrand et al. displayed the limited value of bitewing radiographs for incipient occlusal caries^9^.

Recently, the literature on MRI sequences has substantially grown, and its application continues to expand. Technical progress, combined with the possibility of radiation-free imaging, greatly increased the interest in MRI use in dentistry^10–15^. The scope of potential MRI applications in dentistry now covers a broad range, from wisdom tooth extraction, implant insertion, periodontal evaluation, and even orthodontics^16–20^. MRI has considerably better soft tissue contrast than radiation-based imaging techniques, and recently, its depiction of dental and osseous structures has become comparable to CT and CBCT with high dimensional stability^21–23^.

The assessment of carious lesions using water-sensitive MRI sequences has not yet been performed. Since salivary penetration of dental hard tissue precedes demineralization and structure loss, early detection of pathological fluid concentration within dental hard tissue would be of high interest. The purpose of this study was to correlate carious lesions, as determined by dental radiographs and OPT, with findings on MRI sequences. A secondary aim was to assess the value of a water-sensitive Short tau inversions recovery (STIR) sequence in patients with clinically verified carious lesions.

## Methods

### Study design

This retrospective cohort study was designed in accordance with STROBE guidelines^24^. All 42 patients who presented at the Department of Periodontology, Ludwig-Maximilians-University Munich and received an MRI at the Department of Diagnostic and Interventional Neuroradiology, Klinikum rechts der Isar, Technische Universität München between October 2018 to April 2019, were retrospectively screened according to the following inclusion criteria:

1. Caries defined as the localized destruction of tooth structure according to Gibbons et al.^25^, which can present as distinct surface changes of the enamel (brown and white lesions) or a cavitation in later stages, and can proceed to pulpal infection if not treated^26^.
2. Imaging quality of Likert Grade ≥ 4 in the affected tooth

**Figure 1:**
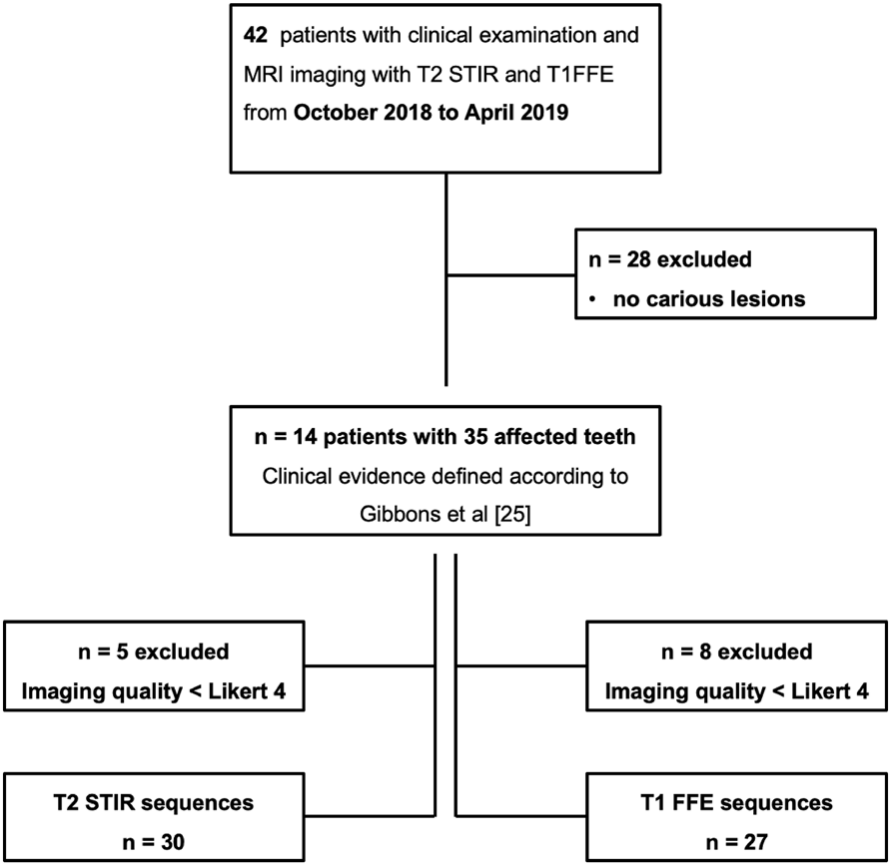
Flow chart shows patient recruitment.

A consort scheme of patient inclusion is shown in Fig. 1. Patient characteristics are summarized in Table 1.

**Table 1:** Patient characteristics.

Patient characteristics (n = 14)	
Age	52 (15)
Age range	20–71
Female	36%
Image quality (Likert grading scale)	4.44 (0.49)
Teeth excluded (artefacts) in STIR/T1 FFE	14% / 23%

Data in parenthesis are SD.

### Clinical examination

All patients underwent standardized dental exam before imaging. Results were documented in standard fashion: Each tooth was categorized into 4 to 5 regions: Buccal, lingual, distal and mesial regions were documented for every tooth. In premolars and molars, an occlusal region was also documented. In each region, fillings and/or the occurrence of carious lesions were documented.

### MRI acquisition

All subjects were scanned with a 3T MRI scanner (Elition, Philips Healthcare, Best, The Netherlands) at the Department of Diagnostic and Interventional Neuroradiology, Technical University of Munich, using a 16-channel head-neck cervical spine array. Patients were positioned head-first in a supine position. The sequence protocol consisted of a short survey scan for sequence position planning (acquisition time 0:39 min), a three-dimensional (3D) isotropic short tau inversion recovery (STIR) sequence (acquisition time 6:03 min, acquisition voxel size 0.65 × 0.65 × 0.65 mm^3^, repetition time (TR) 2300 ms, echo time (TE) 184 ms, inversion recovery (IR) 250 ms, compressed sense, reduction 5, gap − 0.5 mm, slice oversampling 1.5, water-fat shift (pix)/bandwidth (Hz) 1766/246), and a 3D isotropic T1-weighted fast field echo (FFE) black bone sequence (acquisition time 5:31 min, acquisition voxel size 0.43 × 0.43 × 0.43 mm^3^, TR 10 ms, TE 1.75 ms, compressed sense, reduction 2.3, gap – 0.25 mm, water-fat shift (pix)/bandwidth (Hz) 1503/289).

### Image analysis

Technical image quality was assessed using a 5-point Likert rating scale (1 = extremely poor, images are not clinically useful; 2 = poor, clinical use is not advised; 3 = average, borderline clinical use due to the image quality; 4 = good, containing no substantial adverse effect for clinical use; 5 = excellent, no restrictions for clinical use). Teeth were excluded from analysis if image quality was < Grade 4, which is defined as image quality with artifacts with little impact on image diagnosis, moderate sharpness and well distinguishable lesions with poorly defined edges^27^.

All clinically affected teeth were included in the analysis. As healthy control, the correlating tooth in the opposite location in the same jaw (i.e., 27 for 17) was selected. If this tooth was extracted, affected by caries, or not analyzable due to image quality of Likert Grade < 4, the proximal tooth was used. 3D sequences were reconstructed in sagittal and coronal planes and aligned to the long axis for every tooth. As additional reference, muscular signal intensity was measured using the masseter muscle.

**Figure 2:**
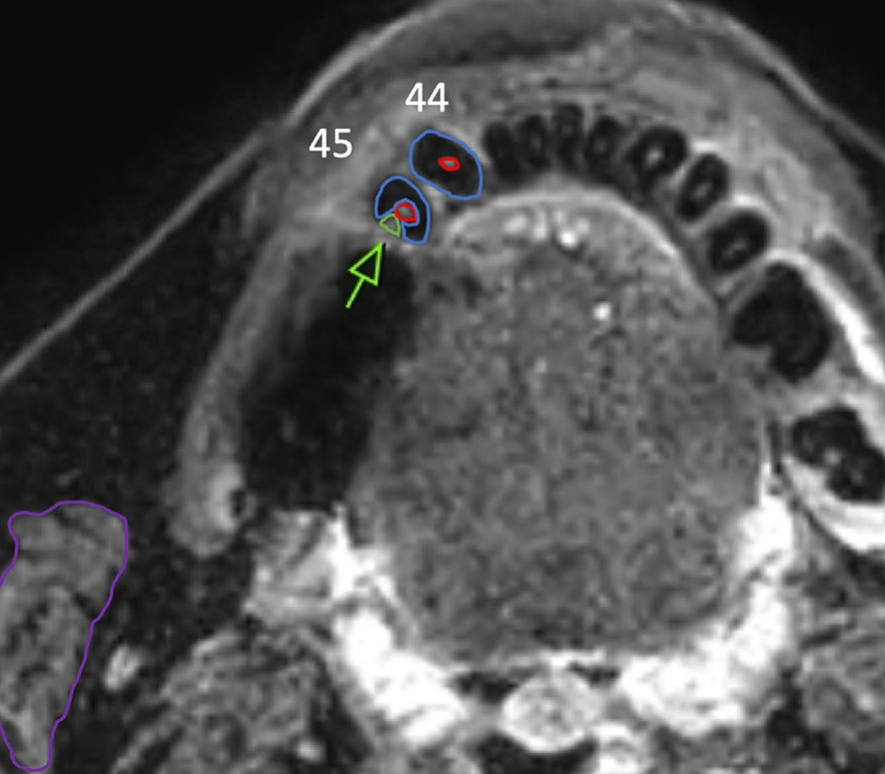
This figure illustrates the ROI placement in a STIR image in a carious tooth 45 and a healthy tooth 44. Purple: masseter muscle, green and arrow: carious lesion, red: pulp cavity, blue: dental hard tissue comprising enamel and dentin.

Apparent signal-to-noise ratios (aSNRs) and aHTMCNR (apparent Hard tissue − muscle contrast-to-noise ratio) were calculated in both sequences with mean signal intensities (SI) and standard deviations (SD) in the selected regions of interest (ROI). ROI conceptualization is exemplarily shown in Fig. 2. This methodology was adapted from measurements of nerve intensities as published for instance by Klupp et al.^28^:

$$aSNR \left(apparent \,signal \,to \,noise \,ratio\right): SIhard\_tissue/SDdhard\_tissue$$

or

$$aSNR (apparent \,signal \,to \,noise \,ratio): SIpulpa/SDpulpa$$

$$aHTMCDR (apparent \,hard\_tissue-muscle \,contrast \,to \,noise \,ratio):$$

$$(SI \,hard\_tissue - SI \,muscle)/SD \,hard\_tissue$$

or

$$aHTMCDR (apparent \,pulpa-muscle \,contrast \,to \,noise \,ratio):$$

$$(SI \,pulpa - SImuscle)/SDpulpa$$

### Statistical analysis

Prism 9 (Graphpad Software LLC, Boston, United States) was used for all statistical tests. Normal distribution was tested using the Kolmogorov–Smirnov test. Wilcoxon matched pairs signed rank test was used to compare aSNR and aHTMCDR values. 20% of all values were tested for intrarater correlation by Pearson r test. P-values of < 0.05 were considered statistically significant.

### Ethics approval and consent to participate

The study received institutional review board approval (Technical University of Munich: Ref.-No.185/18 S and Ludwig-Maximilians-University Munich: Ref.-No. 18-657). All participants gave written informed consent to the study. The authors confirm that all methods were carried out in accordance with relevant guidelines and regulations including the principles described in the Declaration of Helsinki.

## Results

### Patient cohort and clinical findings

Patient characteristics are summarized in Table 1. In this study, 14 patients with a total of 30 carious lesions met inclusion criteria.

### Findings in T1 FFE sequences

**Figure 3:**
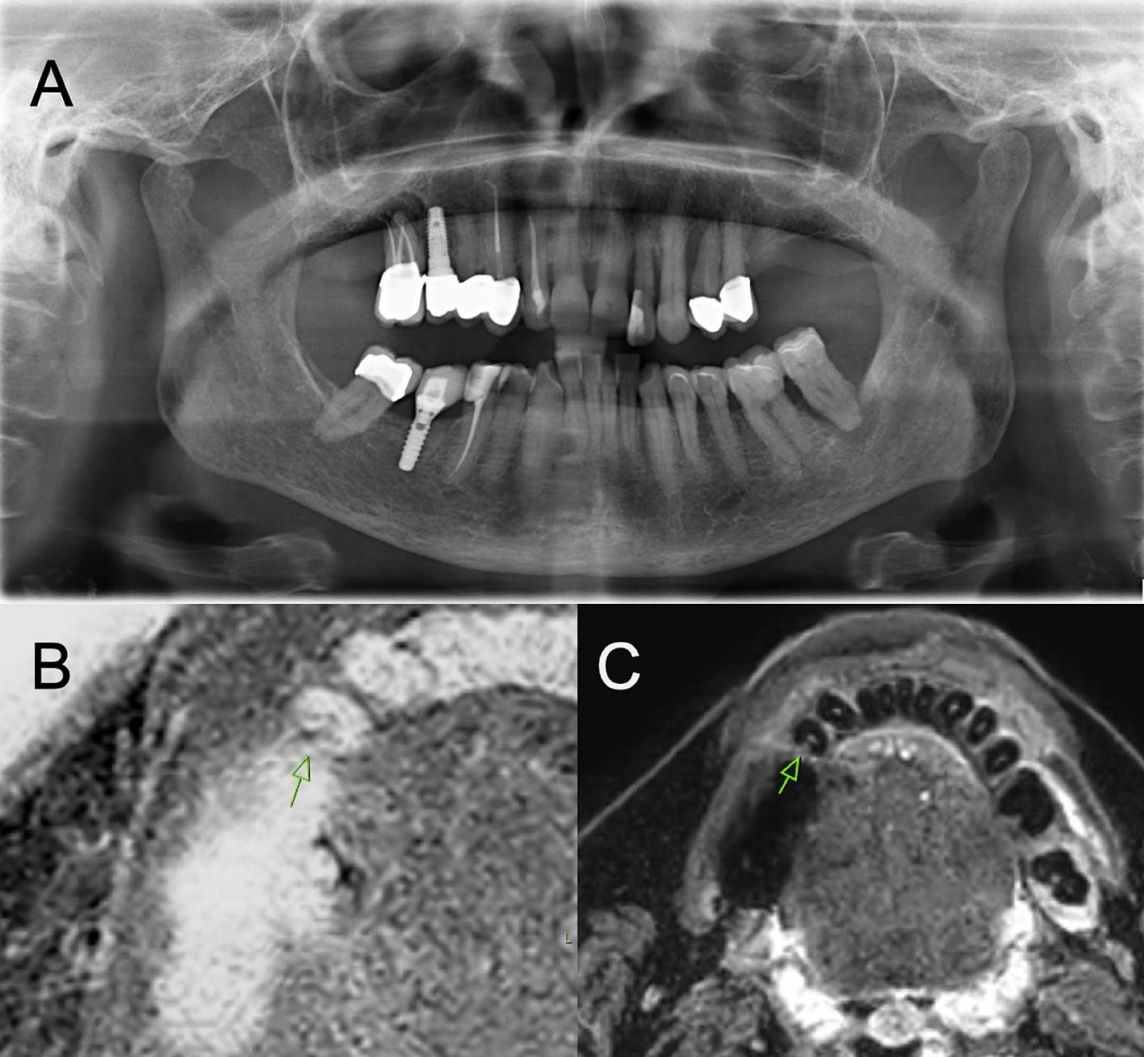
This figure shows a 67 years old female patient with multiple restorations. There are crowns inserted in 45 and 47 but also a titan implant in regio 46. These restorations cause metal artifacts in the corresponding region, but they do not hamper diagnostic accuracy with regard to the caries lesion distal in 44. (**A**) In the OPT a D4 carious lesion distal in the tooth 44 proximal to 45 can be detected, which also could be identified by clinical inspection. (**B**) The axial slice of the T1 FFE sequence shows a deep lesion located distally in 44 with association to the pulp cavity. Comparing to the STIR sequence depicted in C) the T1 FFE sequence is more prone to artifacts caused by metal restorations. C) The STIR sequence shows a hyperintesity with a similar configuration to the T1 based sequence. In both sequences the metallic artifacts caused by the implant placed in regio 46 and the prosthetic restorations do not hamper the diagnostic quality with regard to caries detection.

All clinically suspected carious lesions were imaged using inverted T1 FFE sequences to create a CT-like image impression (Fig. 3). Due to susceptibility motion and metal artifacts reported for this sequence^29^, 3 teeth were excluded due to poor image quality. Quantitative analysis using aSNR ratios showed no significant differences between the pulp of healthy and carious teeth (p = 0.06). Significant differences using aSNR ratios were detected between the dentin in healthy teeth and carious lesions (p < 0.001). Quantification using aHTMCNR also revealed significant differences between dentin in healthy teeth and carious lesions (p < 0.001). All remaining ratios were insignificant.

### Findings in STIR sequences

**Figure 4:**
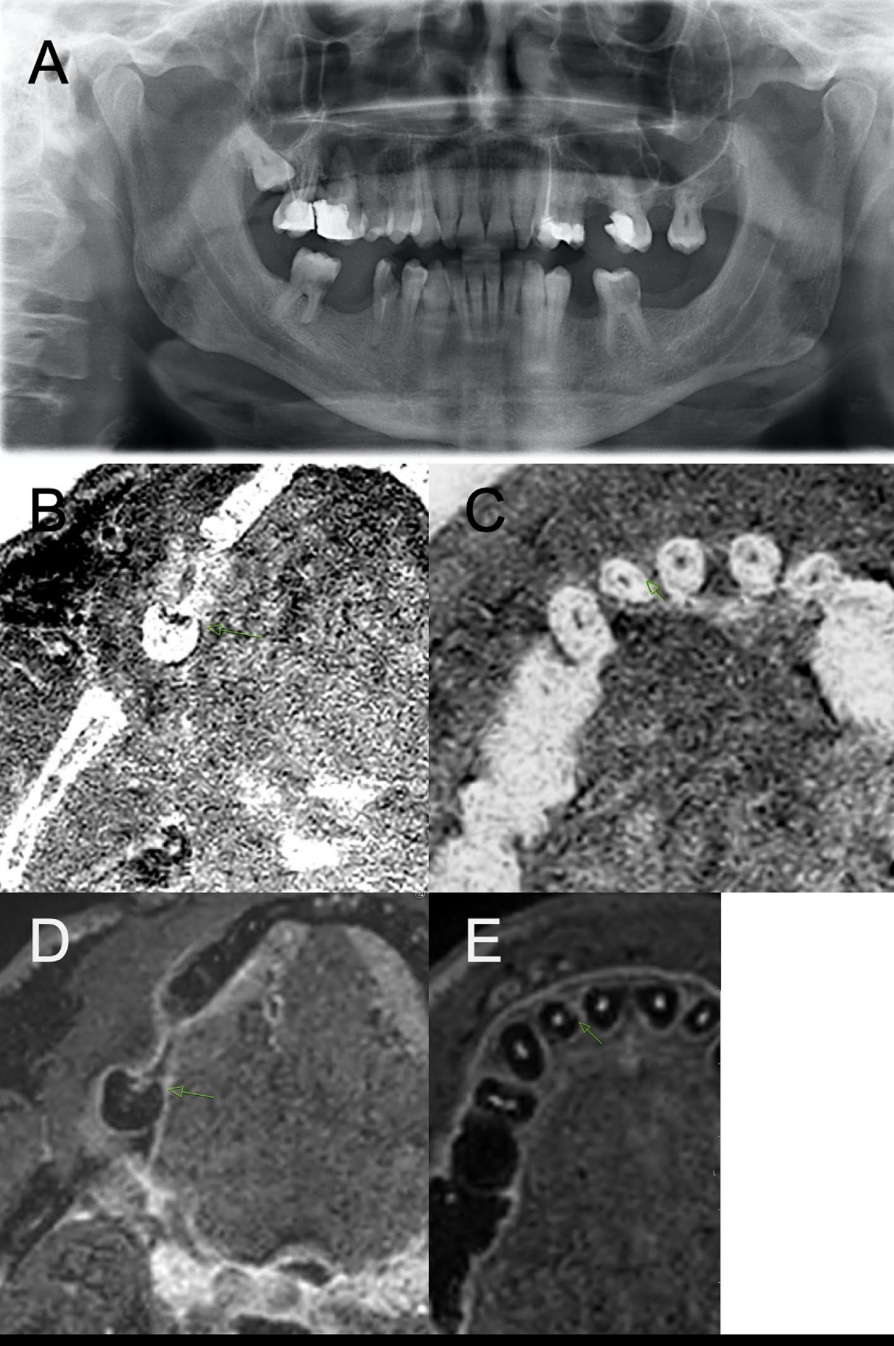
This figure shows a 40 years old male patients with multiple extensive carious lesions in the molar region of the mandible. (**A**) The OPT shows D4 carious lesions in the teeth 36 and 46 also detected clinically and verified by dental radiography (not shown). Also, a carious lesion can be detected in 44, which dispayed only a subte superficial lesion of the enamel but extensive destruction of the dentin. (**B**) In the T1 FFE sequence corresponding to the radiolucency a carious lesion is depicted in 46. (**C**) The STIR sequence also shows a localized hyperintensity with slightly minor dimension. With regard to the size of the carious lesion it can be assumed that only some areas maintain activity whereas some are already in an arrested state. (**D**) The 45° rotated tooth 12 shows no definite pathology neither in T1 based imaging nor in the OPT or clinical inspection. (**E**) In the STIR sequence a subtle localized hyperintensity is visualized in the mesial part of 12 and the gingival tissue between 11 and 12.

In the STIR sequence, carious lesions presented as a hyperintensity compared to the surrounding healthy hard tissue (Fig. 3A–C). The STIR sequence also allowed for visualization of 25 proximal hyperintensities not detected clinically or in OPT (Fig. 4A–E). However, these findings were not statistically significant.

Further, carious lesions showed a high signal heterogeneity. There were significant differences in signal intensities using the aSNR ratios between the healthy tooth and carious esions (p < 0.001) as well as between the hard tissue and pulp in the carious and healthy teeth (p = 0.03 and p = 0.02 respectively). Comparisons of aHTMCNR ratios revealed a significant difference between pulp signal in carious teeth and healthy teeth (p = 0.01). Also, the signal to noise ratio of carious lesions was significantly higher than of healthy hard tissue (p < 0.001) (please see Tables 2 and 3).

**Table 2:** Results of T1 FFE imaging.

aSNR (n = 27)	Healthy tooth	Carious tooth	p
Hard tissue	3 [2, 4]	3 [2, 3]	0.63
Pulp	9 [6, 13]	9 [6, 14]	0.06
Carious lesion	–	4 [2, 6]	< 0.001*
aHTMCNR (n = 27)			
Hard tissue	− 24 [− 29, − 12]	− 20 [− 29, − 12]	0.865
Pulp	− 1 [− 64, 0]	− 0 [− 3, 2]	0.29
Carious lesion	–	− 11 [− 15, − 6]	< 0.001*

Data in parenthesis are IQR.

*aSNR* apparent signal-to-noise ratio, *aHTMCNR* apparent hard tissue—muscle contrast-to-noise ratio.

*Carious lesion vs. healthy hard tissue of the same tooth. Significant values are in bold.

**Table 3:** Results of STIR imaging.

aSNR (n = 30)	Healthy tooth	Carious tooth	p
Hard tissue	5 [5, 12]	5 [4, 6]	**0.03**
Pulp	17 [12, 33]	14 [10, 18]	**0.02**
Carious lesion	–	8 [5, 12]	** < 0.001**
aHTMCNR (n = 30)			
Hard tissue	− 16 [− 40, − 12]	− 14 [− 23, − 10]	0.10
Pulp	10 [7, 21]	9 [5, 12]	**0.01**
Carious lesion	–	− 8 [− 12, − 4]	** < 0.001**

Data in parenthesis are IQR.

*aSNR* apparent signal-to-noise ratio, *aHTMCNR* apparent hard tissue—muscle contrast-to-noise ratio.

*Carious lesion vs. healthy hard tissue of the same tooth. Significant values are in bold.

### Interreader agreement

Ratings were done by two independent, blinded readers. Interreader reliability was high (r = 0.78, p < 0.001).

## Discussion

In this study, T1 based imaging identified statistically significant differences in signal intensities between hard tissue (comprising enamel and dentin) of healthy teeth compared to carious teeth. The results of this study validate prior work on T1 based imaging using ultra short echo times^5,30^. In addition, the use of a water-sensitive STIR sequence allowed for detecting significantly lower pulp signal to noise ratios in carious teeth but also significantly higher signal to noise ratios in carious lesions compared to healthy dental hard tissue. Another finding was, that signal intensities of carious lesions in STIR sequences were more heterogenous compared to T1. This observation could mirror the various levels of disease activity of in-vivo carious processes. However, it has to be stated that until today there are no reproducible and generally applicable rules to determine the precise level of activity within carious lesions. In dental offices clinical judgement and subjective measures are used, which are based on the dentists’ experience. More active lesions harbor a moist, white surface with softer dental matrix, whereas arrested caries appears darker and harder^8,31^.

Recent studies on the use of dental MRI has included fields of periodontology^10,16,32^, endodontology^21,33^, implantology^18,19^, traumatology^34,35^ and orthodontics^20^. Dental MRI has also investigated pulpal reactions to deeply penetrating carious lesions using ADC maps and T2 mapping, however, dentists have been skeptical regarding its clinical implementation^7,36,37^. With regard to image quality of the acquired images, the use of intra-oral coils specifically designed for dental applications allowed for taking a step further to optimizing diagnostic accuracy. Recently, the transition from regular MRI to dental dedicated MRI is fostered scientifically and clinically by Hilgenfeld et al. and Al-Haj Husain et al. showcasing the potential of the technique for different application fields like preoperative imaging and endodontics^21,22,38^. The lack of ionizing radiation associated with MRI has made it in extremely attractive alternative to traditional radiation-based imaging. Recent research has investigated the use of ultra-short echo time sequences to achieve a comparable spatial resolution like X-ray or CT using MRI, and thereby avoiding potential negative side effects of ionizing radiation^39–41^. The same efforts have been made recently in cariology, with the intent to generate images harbouring information similar to bitewing imaging or OPT^5,42^.

The caries process has been studied since the fifteenth century, and it remains a critical interest in dental research today^43^. Although the bacterial toxin related decrease in pH and its induction of demineralization has been elucidated, the difference between active and arrested carious lesions remains a major issue in dental diagnostics today^8,9,25,26,44^. Clinically, caries detection is based on visual inspection of an air-dried lesion followed by palpation with a ball-ended explorer^26,44^. In meta-analyses the use of different activity indices has been evaluated, amongst which the International Caries Detection Assessment System (ICDAS) and Nyvad scoring system are the best known, and guide decision making on whether to excavate a carious lesion or not^26,44–46^. However, both of these scoring systems are based on clinician experience and evaluation, and therefore subjective assessments. In addition, proximal tooth lesions cannot be visually inspected, which increases the risk of missing subtle lesions. In these cases, bitewing radiographs are needed. However, as discussed above, these traditional imaging techniques cannot detect early caries process prior to demineralization^2^. There are recent scientific efforts investigating optical methods for the assessment of proximal carious lesions^3,4,47^.

As displayed in this study, the use of a water-sensitive STIR sequence could be the newest addition to the armamentarium in the assessment of proximal carious lesions. In addition, our study found a high signal heterogeneity within the carious lesions, potentially mirroring different stages of activity associated with varying localized fluid leakage. The presented results and images allow for a first glimpse of what could be feasible in the future. However, prospective studies, including a larger cohort with clinical caries activity assessment and subsequent correlation with MRI are needed. The results of this study found that both sequences, the T1 gradient-echo and the STIR sequence, allow for the detection of caries in vivo with good correlation to conventional imaging and inspection.

This study has several limitations. First, the retrospective study design is prone to selection bias. Second, the small number of included patients does not allow for further conclusions with regard to the majority of patients. Third, no dedicated intraoral coil was used, which was reported before to have significant beneficial effects on image quality^21,22,33,38^. Further limitations are that MRI analysis was conducted on carious lesions detected on dental x-ray, the detection accuracy of MRI for dental caries has not yet assessed. The scanning time of the applied protocol was approximately 12 min, which is reduced greatly to less than 10 min by novel MR sequence protocols. Reducing the scan time is inherently associated with increasing image quality. Another limitation is, that the majority of lesions assessed by this study are severe carious lesions that can be detected clearly on dental x-rays. Furthermore, as shown by Fig. 4 there are lesions with a minor surface defect and a large deep carious lesion, which is easily identified by x-ray but is hardly detected using MRI. It also has to be mentioned, that metallic and movement artifacts can cause severe impairment of diagnostic quality in the applied sequence protocol reducing the number of included patients. Last, the retrospective nature of this study did not allow for correlation of pathological signal alterations in the MRI with supposedly negative clinical findings but only with x-ray records.

## Conclusion

Using T1 gradient-echo and STIR sequences for caries detection was feasible and accurate in this clinical sample. The STIR sequence might offer advantages compared to conventional radiation-based dental imaging and clinical inspection with regard to detection of clinically asymptomatic lesions with unknown significance. Further prospective studies are needed to evaluate the use of water-sensitive MRI-sequences for early caries detection compared to visual inspection combined with x-ray imaging.

## Data Availability

All source data are stored at the Department of Neuroradiology, Technical University of Munich, Munich, Germany. We invite parties interested in collaboration and data exchange to contact the corresponding author directly.
